# *Antarctolichenia onofrii* gen. nov. sp. nov. from Antarctic Endolithic Communities Untangles the Evolution of Rock-Inhabiting and Lichenized Fungi in *Arthoniomycetes*

**DOI:** 10.3390/jof7110935

**Published:** 2021-11-03

**Authors:** Lucia Muggia, Claudia Coleine, Roberto De Carolis, Agnese Cometto, Laura Selbmann

**Affiliations:** 1 Department of Life Sciences, University of Trieste, Via Giorgieri 10, 34127 Trieste, Italy; lmuggia@units.it (L.M.); roberto.decarolis@phd.units.it (R.D.C.); agnese.cometto@phd.units.it (A.C.); 2 Department of Ecological and Biological Sciences, University of Tuscia, Largo dell’ Università, 01100 Viterbo, Italy; coleine@unitus.it; 3 Mycological Section, Italian Antarctic National Museum (MNA), 16128 Genoa, Italy

**Keywords:** algae, *Lichenostigmatales*, melanization, microbial communities, phylogeny, *Stichococcus*

## Abstract

Microbial endolithic communities are the main and most widespread life forms in the coldest and hyper-arid desert of the McMurdo Dry Valleys and other ice-free areas across Victoria Land, Antarctica. There, the lichen-dominated communities are complex and self-supporting assemblages of phototrophic and heterotrophic microorganisms, including bacteria, chlorophytes, and both free-living and lichen-forming fungi living at the edge of their physiological adaptability. In particular, among the free-living fungi, microcolonial, melanized, and anamorphic species are highly recurrent, while a few species were sometimes found to be associated with algae. One of these fungi is of paramount importance for its peculiar traits, i.e., a yeast-like habitus, co-growing with algae and being difficult to propagate in pure culture. In the present study, this taxon is herein described as the new genus *Antarctolichenia* and its type species is *A. onofrii*, which represents a transitional group between the free-living and symbiotic lifestyle in *Arthoniomycetes*. The phylogenetic placement of *Antarctolichenia* was studied using three rDNA molecular markers and morphological characters were described. In this study, we also reappraise the evolution and the connections linking the lichen-forming and rock-inhabiting lifestyles in the basal lineages of *Arthoniomycetes* (i.e., *Lichenostigmatales*) and *Dothideomycetes*.

## 1. Introduction

The ice-free zones of continental Antarctica, which include the peaks of the Transantarctic Mountains emerging from the Polar Plateau and the widest area of the McMurdo Dry Valleys, are the driest, coldest, and most remote environment on Earth. In the McMurdo Dry Valleys in particular, annual snowfalls range from 3 to 50 mm (water equivalent only) [1], which decreased by 1 mm through 2017 [2]; water rarely reaches the ground as a few drops, while mostly sublimes, or is blown away [3]. In certain locations, precipitations have been lacking for nearly two million years (Ma). In these regions, accounted as the Mars analogue on our planet, the environmental conditions reach the limits for supporting life in terms of low temperature, oligotrophy, and aridity. They have been considered to be devoid of life for a long time, until microbial communities were discovered as dwelling inside rocks, finding an ultimate refuge in the more buffered conditions offered by the endolithic niche [4,5]. In this environment, endolithic microbes represent one of the most widespread life forms and the main standing biomass [6,7,8]. The ability to exploit the endolithic niche is, therefore, a key adaptation for microbes to successfully reproduce and spread in the Antarctic desert.

Among the Antarctic endolithic communities described, the most studied and widespread are those dominated by lichens occurring both cryptoendolithically, mainly in sedimentary rocks (i.e., sandstones [9,10]), and chasmoendolithically, mainly in granite systems [11]. These are complex and self-supporting assemblages of phototrophic and heterotrophic microorganisms, including bacteria, chlorophytes, and both free-living and lichen-forming fungi. Chlorophycean (lichenized and free-living algae) and cyanobacteria are the only primary producers in these niches [3]. Lichens, in particular, show a peculiar morphological adaptation, whereby they give up their typical thallus organization and grow simply as filamentous forms to more efficiently colonize the thinnest airspaces of porous rocks [3,4]. In the endolithic niche, the lichen-forming fungi still clearly develop haustoria and appressoria to assure the specific relationship with their compatible algal partner. The adaptation to a loose morphological fungal–algal relationship may have promoted the key transition between the free-living and the fully lichenized lifestyles. The most frequent lichenized fungi in the Antarctic cryptoendolithic communities belong to the orders *Lecanorales* and *Lecideales* and, to a lesser extent, to *Acarosporales* and *Caliciales* [12,13]. This is not surprising since *Lecanora fuscobrunnea* and *Lecidea cancriformis* (*Lecanorales* and *Lecideales*, respectively) are the two most widespread endemic species in continental Antarctica [14,15]. These species are especially dominant in the dry Antarctic areas, in which the presence of other lichens, such as *Buellia grisea*, *B. pallida*, and *Carbonea capsulata* has been reported. Lichenized fungi, mainly in the genus *Acarospora* (e.g., *Acarospora gwynii*), are also frequently retrieved from these communities [12,16] and, recently, *L. fuscobrunnea* was obtained in pure culture (data unpublished). 

A fungus with peculiar characteristics has been recurrently isolated (first isolate MNA-CCFEE5176, [17]) in over approximately 25 years of culture-based studies, undertaken on a wide selection of colonized rocks collected during numerous sampling campaigns in Antarctica. Its nuclear internal transcribed spacer (nucITS) sequence showed an identity of 87–88% in GenBank, with sequences barely related to the lichenized order *Arthoniales*, while, in a multilocus phylogeny, this fungus was placed close to the lichenicolous genus *Lichenostigma* [18]. This fungus appeared new also for morphological and cultural characteristics, having a yeast-like habitus and was difficult to propagate in pure culture, possibly suggesting that it represented a transitional group between a free-living and symbiotic lifestyle in the *Arthoniomycetes*. To support this hypothesis, we obtained morphological and sequence data from additional isolates and compared them with molecular data retrieved from public databases. 

In the present study, we delineated (i) the phylogenetic placement of the new taxon analyzing three molecular markers; (ii) the morphological characteristics to explain the degree of the relationship with neighboring phylogenetic lineages in an evolutionary context; and (iii) the ecology of the fungus while considering the connections established with algae, to clarify their degree of interaction/interdependence. Along with the novelty of this phylogenetic fungal group and its description, we also studied the taxonomy of the associated algae and presented our considerations on the evolution of the different lifestyles and the potentiality of lichenization in the basal lineages of *Arthoniomycetes*.

## 2. Materials and Methods

### 2.1. Study Area and Rock Sampling 

**Figure 1:**
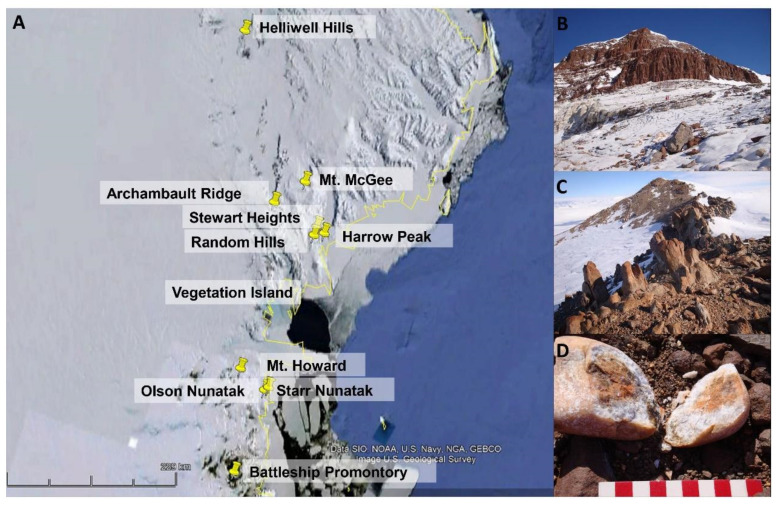
(**A**) Map of Victoria Land in continental Antarctica and the localities from where *Antarctolichenia onofrii* strains have been isolated; (**B**,**C**) a typical environment characterizing the sampling localities: the Archambault Ridge (**B**) and Random Hills (**C**); and (**D**) the rock colonized by cryptoendolithic communities on Mt. Howard.

Endolithically colonized rocks were collected in eleven different localities in Victoria Land (continental Antarctica), during the Italian Antarctic Expeditions (December 2004 and December 2010–January 2011) and the Ganovex XI Expedition (the Dutch Antarctic Expedition of the German Antarctic North Victoria Land Expedition project, December 2015–January 2016) (Figure 1, Table 1).

The direct observation, in situ, using magnifying lenses assessed the presence of endolithic colonization. Rock samples were aseptically excised using a geological hammer, collected in plastic sterile bags, transported, and then stored at −20 °C at the University of Tuscia (Italy), until downstream analyses were performed.

### 2.2. Fungal Isolation and Molecular Identification

Fungal isolation from rocks were performed by grinding the samples and fragments were inoculated (in duplicate) in Petri dishes filled with 2% malt extract agar (MEA, AppliChem, GmbH, Darmstadt, Germany). Growth media were supplemented with chloramphenicol (100 ppm) to prevent bacterial growth. Plates were incubated at 15 °C and inspected on a weekly basis until no new fungal colonies appeared. The colonies were then transferred to MEA slant tubes and incubated at 15 °C, from weeks to a few months, to obtain a sufficient biomass for DNA extraction. 

DNA was extracted using the Nucleospin Plant kit (Macherey-Nagel, Düren, Germany). The nucITS was amplified using the primers ITS5 and ITS4 [19]. The nuclear ribosomal large subunit (nucLSU) was amplified using the primers LR0R and LR7, while the nuclear ribosomal small subunit (nucSSU) was amplified uisng the primers SR1R and NS8 ([20], http://www.biology.duke.edu/fungi/mycolab/primers.htm, accessed on February 2021). Polymerase chain reactions (PCRs) were performed using BioMix (BioLine GmbH, Luckenwalde, Germany). The PCR mixtures were prepared with 5 pmol of each primer and 20 ng of template DNA; Milli-Q sterile water was added to a final volume of 25 µL. Amplification was carried out using a MyCycler™ Thermal Cycler (Bio-Rad Laboratories, GmbH, Munich, Germany). 

The PCR protocol was as follows: 3 min at 95 °C for the first denaturation step; then 35 cycles of a denaturation step at 95 °C for 30 s; followed by an annealing step at 55 °C for 30 s for the ITS region; an annealing step at 52 °C for 30 s for the nucLSU–nucSSU regions; and an extension step at 72 °C for 30 s. The last extension was at 72 °C for 5 and 7 min for the nucITS and the nucLSU–nucSSU regions, respectively. All the amplicons were checked for their quality and size using 1.5% of agarose gel electrophoresis stained with GelRed™ (Biotium, Fremont, CA, USA), and purified using a Mag-Bind^®^ Normalizer Kit (Omega Bio-Tek Inc, Norcross, GA, USA). Purified amplicons were sequenced with the same PCR primers using Macrogen Inc. (Seoul, Korea). The sequence assembly was performed using the ChromasPro v.1.32 software (Technelysium, Southport, Queensland, Australia) and sequences were compared in the GenBank (NCBI) database using BLASTn [21]. 

All the fungal strains obtained were both cultivated on slant agar (MEA) in glass tubes (15 × 2 cm) and cryopreserved in a metabolically inactive state at −150 °C in the the Italian National Antarctic Museum—Culture Collection of Fungi From Extreme Environments (MNA-CCFEE).

### 2.3. Algal Isolation and Molecular Identification

Algal colonies were noted to develop and co-grow within the fungal biomasses of two isolates, MNA-CCFEE6564 and MNA-CCFEE6583, and were isolated axenically. Algal cells were transferred and further subcultured axenically on new agar plates containing Bold’s Basal medium (BBM, [22]), a malt yeast extract medium (MY, [23]), and the Trebouxia medium (TM, [22]). Algal DNA was extracted following the cetyltrimethyl ammonium bromide (CTAB) protocol by Cubero et al. [24] and amplified using the universal primers LR7 and LR0R [20] for the nucLSU, and rbcL320 and rbcL803 primers [25] for the plastidial locus coding the ribulose-1,5-bisphosphate carboxylase large subunit (rbcL) using the PCR condition as in Muggia et al. [26].

### 2.4. Phylogenetic Analyses 

After checking the identity of the fungal sequences of the three sequenced loci (nuclear ITS, LSU, and SSU; Table 1) by blast search in GenBank, the phylogenetic position of the strains was initially studied using a comprehensive taxon sampling, including representative taxa of *Eurotiomycetes*, *Dothideomycetes*, and *Arthoniomycetes* for each individual locus of nucITS, nucLSU, and nucSSU (Supporting Table S1). In this selection, we considered both lichenized and non-lichenized fungi, as well as basal lineages in *Arthoniomycetes* and *Dothideomycetes*, which represent the borderline lichens. The fungal taxa have been selected according to previous phylogenetic studies [18,27,28,29,30,31,32,33,34] and their sequences were downloaded from the NCBI GenBank (Supporting Table S1). Due to missing sequence data in GenBank for many taxa, which caused inconsistency among the nucITS and the other two loci, we decided to follow first a consensus phylogenetic approach in which the three sequenced loci were analyzed individually. We kept the nucITS locus dataset alone, as its taxon sampling (due to availability of sequence data in GenBank) substantially differed from the nucLSU and nucSSU datasets. In particular, nucITS sequence data are lacking for taxa belonging to *Lichenostigmatales*. Having verified the topological consistency between the nucLSU and the nucSSU, we decided to combine these two datasets in a concatenated analysis. Having confirmed the placement of the new sequences within *Arthoniomycetes*, we used representatives of *Dothideomycetes* as outgroups and proceeded with the concatenation approach for the nucLSU and nucSSU datasets. The combined nucLSU-nucSSU dataset was implemented to include all representative species and relative type species within *Arthoniomycetes* to improve the resolution of the new taxon within this class. 

A total of 157 taxa were included in the combined nucLSU-nucSSU dataset, which were representatives of *Lichenostigmatales* (with the single family *Phaeococcomycetaceae*), *Arthoniales* (with the families *Arthoniaceae*, *Chrysothricaceae*, *Lecanographaceae*, *Opegraphaceae*, *Roccellaceae* and *Roccellographaceae*), and *Dothideomyceta* (with the order *Collemopsidiales*); additional eight species of *Dothideomycetes* were selected as outgroup (Supporting Table S1).

Similarly, the identity of the algal strains was checked by blast search [21] in GenBank, and the closest hits were selected for the phylogenetic analyses. Other algal sequences were selected from previously published phylogenies [35,36] and downloaded from GenBank. The two sequenced loci nucLSU and plastidial rbcL were analyzed individually due to the lack of sequence data for both loci for each algal taxon. The nucLSU algal sequence dataset included a broad spectrum of taxa belonging to the classes *Trebouxiophyceae* (with the orders *Chlorellales*, *Prasiolales* and *Trebouxiales*) and *Chlorophyceae*, while two species of *Chlorodendrophyceae*, i.e., *Tetranselmis striata* and *T. suecica* represented the outgroup. The rbcL dataset was restricted to the class *Trebouxiophyceae* and three species of *Prasiolales* were selected as outgroup, i.e., *Prasiola furfuracea*, *P. linearis* and *P. meridionalis* (Supporting Table S2).

The preparation of the multiple sequence alignments (MSA) for each individual locus of either the fungal and algal datasets was performed with clustalW using BioEdit v.7.2 [37], while the combined fungal dataset (nucLSU-nucSSU) was prepared with SequenceMatrix v.1.7.8 [38]; introns and ambiguous regions were not included in the analyses. The phylogenetic analyses were performed for the fungal and the algal datasets using the maximum likelihood (ML) approach [39,40] as implemented in RAxML v.8.2.10 [41], applying the GTRGAMMA model and running 1000 bootstrap replicates. For the fungal dataset, we also run a Bayesian analysis with the program MrBayes v3.2.5 [42]. Two runs of four simultaneous Markov chains were run for 2,000,000 generations and trees were sampled every 100th generation. The distribution of log-likelihood scores was examined using the program Tracer v.1.5 [43] to determine the stationary phase for each search was reached and chains had achieved convergence. The first 25% of the sampled topologies were discarded as part of a burn-in procedure, while the remaining trees were used for calculating the posterior probabilities in the majority rule consensus tree. The convergence of the chains was also confirmed by the convergent diagnostic of the Potential Scale Reduction Factor (PSRF), which approached 1 [42]. The phylogenetic trees were visualized in TreeView v1.6.6 [44].

### 2.5. Morphological Analyses

The analyses of morphological and anatomical characters have been performed on five fungal strains that represent the newly recognized lineage, i.e., MNA-CCFEE5176, MUT 6405 (=MNA-CCFEE6102), MNA-CCFEE6163, MNA-CCFEE6574 and MUT 6552 (=MNA-CCFEE6564) which was selected as holotype. The strains were analyzed using standard microscopic techniques and documented with digital photographs. Analyses and photographs were performed on subcultures that were approximately one year old, and the following characteristics were considered: the form of growth (filamentous vs. yeast-like); the melanization of the hyphae; the form and size of the hyphal cells; the branching of the hyphae; the development of conidiogenous cells; and the formation of conidia. Small fragments of mycelia were removed, and squashed sections were mounted in water and studied using light microscopy. Morphological analyses were performed on the two algal strains that were axenically isolated from the fungi MNA-CCFEE6564 and MNA-CCFEE6583 and considered both the form and size (length/width) of the cells. 

All images were acquired with a ZeissAxioCam MRc5 digital camera that was fitted to the microscope, digitally processed, and slightly refined in sharpness and color tone using Adobe Photoshop 7.0. The figures were prepared using CorelDRAW X4.

## 3. Results

### 3.1. Fungal Isolation and Molecular Identification

We obtained a total of 14 fungal isolates for which we generated 14 new nucLSU, 12 nucSSU and 11 nucITS sequences (Table 1). Over the previous years, seven strains were continuously propagated in culture and stored as cryostocks in the MNA-CCFEE. Two of the strains analyzed here were also deposited in the Mycotheca Universitatis Taurinensis (MUT) collection (MUT 6405 = MNA-CCFEE6102; MUT 6552, holotype = MNA-CCFEE6564). 

**Figure 2:**
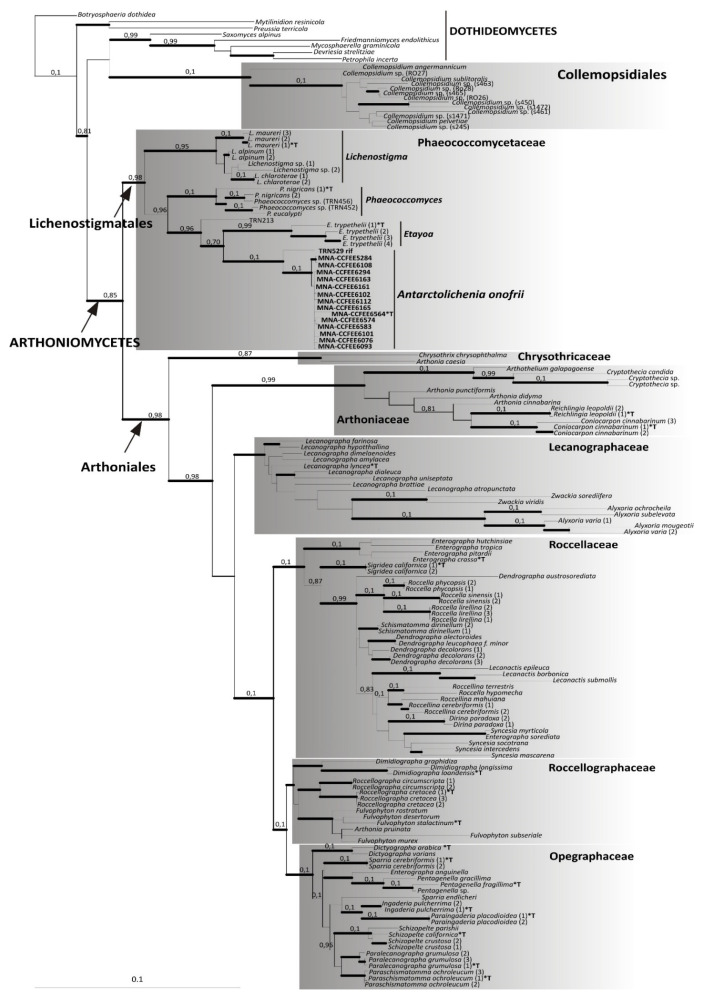
Phylogenetic analysis based on the combined nucLSU and nucSSU sequences of *Arthoniomycetes*, using *Dothideomycetes* as an outgroup. The data for taxa retrieved from GenBank are reported in the Supplementary Table S1 and the different samples of the same species are identified by a numeration in parenthesis after the genus/species name. The maximum likelihood and Bayesian topology are fully consistent. The newly identified lineage of *Antarctolichenia* is highlighted by reporting the strain numbers in bold. The type species included in the analyses are marked by an asterisk and a T (*T). Bayesian posterior probabilities (PP) are reported above the branches and the thickness of the branches highlights those receiving a full ML bootstrap support of 96–100%. The scale bar is proportional to the substitution rate.

The ITS single locus inference congruently resolved the new taxon as a new lineage basal in *Arthoniomycetes (*Supplementary Figure S1), placing the new sequences together with four samples of *Phaeococcomycetaceae* within the *Lichenostigmatales*. *Lichenostigmatales* is a monophyletic and fully supported sister clade of *Arthoniales.* The ML and Bayesian analyses of the combined nucLSU–nucSSU dataset are topologically concordant (Figure 2). Furthermore, this tree topology is concordant with previous phylogenies, including *Dothideomyceta* [18,29,32], and consistently supports the phylogenetic placement of the new taxon as the sister lineage of *Etayoa* (Figure 2). *Dothideomycetes* clearly segregates from *Arthoniomycetes* and the order *Collemopsidiales* is recovered basal to *Dothideomycetes*. Within *Arthoniomycetes*, all orders and families are monophyletic and fully supported (both by bootstrap values and Bayesian posterior probabilities), and their topology is congruent with the phylogenetic inferences previously published by Ertz and coauthors [27,30,31]. Moreover, the genus-based lineages within *Phaeococcomycetaceae*/*Lichenostigmatales*, i.e., *Lichenostigma*, *Phaeococcomyces*, *Etayoa*, and the new taxon are fully supported and individually monophyletic. The phylogenetic analysis places the two Mediterranean rock-inhabiting fungi (TRN213 and TRN529) on individual branches basal to the clades of *Etayoa* and of the new taxon, respectively.

### 3.2. Morphological Analyses of Isolated Fungal Strains

**Figure 3:**
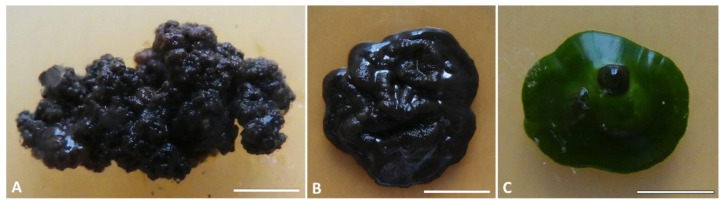
Colony appearance in the culture of *Antarctolichenia onofrii:* (**A**) the isolated strains of MNA-CCFEE6163 and (**B**) MUT 6552 alone, and (**C**) the strain MNA-CCFEE6574 grown together with the Stichococcus-like algae. Strains were grown on MEA (17 °C, 20 μmol fot·m^−2^·s^−1^, with a light/dark cycle of 14/10 h). Scale bars: (**A**) 3 mm; (**B**) 8 mm; and (**C**) 6 mm.

**Figure 4:**
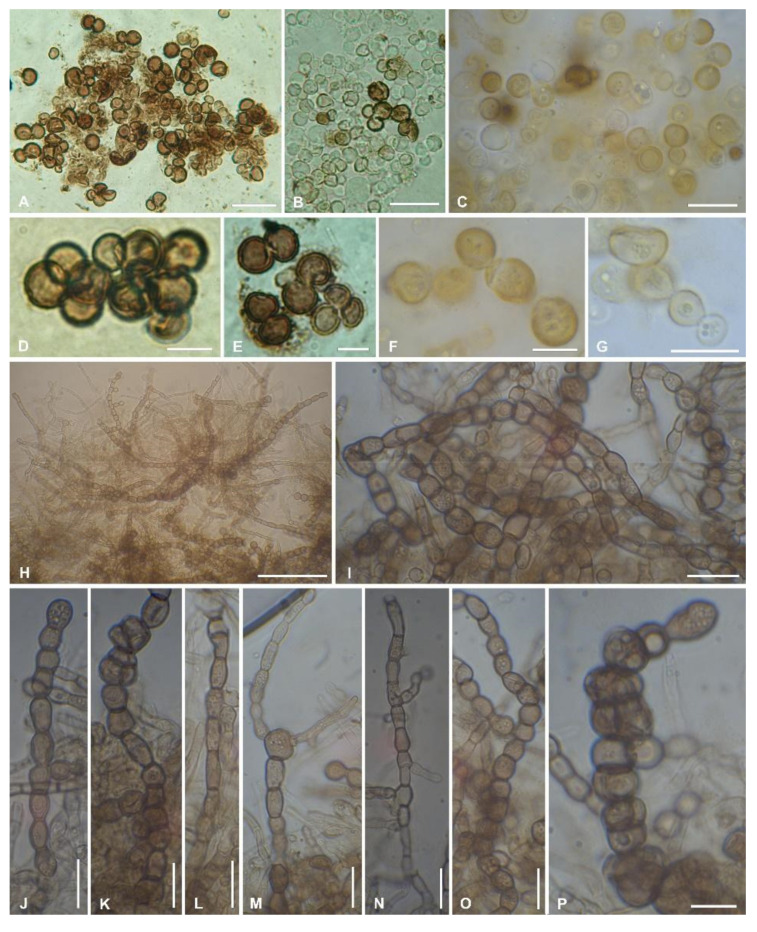
Morphological characters of *Antarctolichenia onofrii* analyzed in the isolated strains MNA-CCFEE5176 (**A**,**B**,**D**,**E**); MUT 6552 (type strain) (**C**); MNA-CCFEE6163 (**F**,**G**); and MUT 6495 (**H**–**P**). Both isodiametric, yeast-like cells with a thick, melanized cell wall (**A**–**G**) and filamentous branching hyphae with a thinner cell wall (**H**–**P**) are observed. The filamentous hyphae are composed of isodiametric-to-rectangular cells (**H**–**P**). Scale bars: (**H**) 50 μm; (**A**,**B**) 20 μm; (**C**,**G**,**I**–**O**) 10 μm; and (**D**,**E**,**F**,**P**) 5 μm.

The new taxon shows a meristematic growth, producing cerebriform colonies (Figure 3A,B) and developing both as filamentous or yeast-like forms (Figure 4). The two mycelium types have been observed in different strains and seem to neither depend on the type of growth medium on which they are propagated/subcultured, nor on the presence of the co-growing algae (Figure 3C). Isodiametric, yeast-like cells present a thick cell wall (Figure 4A–G) which is heavily melanized in more mature cells, while cells are almost hyaline in their young stages (Figure 4B,C,G). The filamentous thallus presents branching hyphae with rectangular-to-oblong cells with a melanized cell wall (Figure 4H–P). Many hyphae also present isodiametric cells, sometimes at the branchings (Figure 4K,M), while others are composed entirely of isodiametric cells (Figure 4O,P). When growing together with the algae, both yeast-like cells and filamentous hyphae developed among the algal cells, but no haustoria-like structures or a more organized mycelium/thallus were observed.

### 3.3. Algal Isolation and Molecular Identification

The sequence analyses of the isolated algae revealed a great similarity of both the nucLSU and rbcL sequences with *Stichococcus bacillaris* (Supplementary Figure S2). Furthermore, the rbcL dataset strengthened the identity of the isolated algae with its geographic origin by presenting its close phylogenetic relationship with *S. antarcticus*. In the rbcL phylogeny, two sequences of *Diplosphaera* retrieved from GenBank, which were the closest blast hits of the isolated algae together with other *Stichococcus* sequences, are nested with *Stichococcus* sequences, suggesting their incorrect identity assignment in GenBank. 

Morphological inspections of the isolated algae confirmed *Stichococcus*-like cells, i.e., elongated cells of about 5–8 μm × 2–3 um wide with one chloroplast, either parietal or placed at one edge of the cell (Supplementary Figure S2C).

## 4. Taxonomy 

***Antarctolichenia*** Selbmann, Muggia, Coleine, ***gen. nov***.—MycoBank: MB839455 (Figure 3 and Figure 4)

*Etymology*: the genus name refers to the geographic origin of the fungus, as it was collected in the Antarctic continent, and to its lifestyle, growing in association with algae, resembling that of a lichen-forming fungus, within endolithic lichen communities.

Monotypic genus in the family *Phaeococcomycetaceae*, order *Lichenostigmatales*, *Arthoniomycetes*, and *Ascomycota*. Endolithic, anamorphic fungus, for which the sexual morph is unknown. Colonies growing rather slowly in vitro and heavily melanized. Thallus composed of yeast-like cells and filamentous hyphae; yeast-like cells with a thick cell wall, heavily melanized, and slightly verrucose in more mature cells, almost hyaline in young stages; filamentous thallus with rectangular cells and more isodiametric cells at the branchings.

Type species: ***Antarctolichenia onofrii*** Selbmann and Muggia **sp. nov.**—MycoBank: MB839456 (Figure 3 and Figure 4).

*Holotype*: MUT 6552 = MNA-CCFEE 6564 cultured strain, isolated from cryptoendolithically colonized sandstone collected in Helliwell Hills, Antarctica. The culture is preserved in a metabolically inactive state at −150 °C.

*Antarctolichenia onofrii* MUT 6552 is the unique identifier of the holotype sheet in the Mycotheca Universitatis Taurinensis (MUT), Department of Life Sciences and Systems Biology, University of Turin. *Etymology*: the species is named after the Italian mycologist Silvano Onofri, who collected the rock sample from which the fungus was isolated for the very first time (Antarctic Expedition PNRA 1996/97).

*Diagnosis*: Strictly rock-inhabiting, endolithic, and asexual fungus. Colonies growing extremely slowly in vitro (reaching about 1 cm in a year), black. A yeast-like and filamentous thallus. Yeast-like isodiametric cells, 5–10 um, with a thick, slightly verrucose cell wall (Figure 4A–G), heavily melanized in more mature cells, and almost hyaline in their young stages (Figure 4B,C,G). Filamentous thallus with rectangular-to-oblong cells, 4–5 × 5–6 um, with a melanized and slightly verrucose cell wall (Figure 4H–P), branching hyphae, isodiametric cells sometimes present at the branchings (Figure 4K,M), and rarely building an entire hyphae (Figure 4O,P). Occasionally growing together with *Stichococcus*-like algae (Supplementary Figure S2C) but not forming haustoria-like or more organized mycelium or lichen-like thallus structures.

*Distribution*: Continental Antarctica, isolated from endolithic lichen-dominated communities in Victoria Land.

*Material examined*: the examined strains and metadata are reported in Table 1.

Notes: as reported for other species in the family *Phaeococcomycetaceae*, *Antarctolichenia onofrii* displays both yeast-like and mycelial organization, conversely, *Phaeococcomyces* spp., reproduced by budding. Unlike *Lichenostigma*, conidiomata and ascomata were not observed in *Antarctolichenia*. *Antarctolichenia onofrii*, is also peculiar for its distribution and ecology, occurring exclusively in lichen-dominated endolithic communities in continental Antarctica.

## 5. Discussion

### 5.1. The Lichen–RIF Connections 

The newly described lineage of *Antarctolichenia* represents an evolutionary connection between non-lichenized and lichen-forming fungi and, interestingly, it emerges within the order *Lichenostigmatales* in the class *Arthoniomycetes*, one of the widest classes of lichen-forming and non-lichenized fungi in *Pezizomycotina* [45,46,47,48,49]. Within *Arthoniomycetes*, *Arthoniales* was the first, well recognized order; it is known to host the highest diversity of mainly corticolous (epiphytic) taxa, forming lichen symbioses with trentepohlioid algae from the tropics [27,50,51,52] and lichenicolous fungi, mostly highly host-specific and commensal on lichens [53]. Furthermore, the existence of *Lichenostigmatales* was first suggested by the phylogenetic studies of Ruibal et al. [54]. The authors, while studying rock-inhabiting, microcolonial fungi (RIF) from the Mediterranean region, identified a group of several rock isolates as *Phaeococcomyces* spp. or *Phaeococcomyces*-like species, but left this lineage unnamed. Regarding this unnamed group, which at that time was placed at the base of *Dothideomycetes*, the authors suggested that it could represent an example of an early diverging lineage of that class [54]. At the same time, several multilocus phylogenetic studies, which also included diverse fungal classes, supplied a strong support for the sister relationship between *Arthoniomycetes* and *Dothideomycetes* [46,47,55]. Thus, the clade grouping of these two big classes (that does not include the unnamed group of Ruibal et al.) was defined as the rankless taxon ‘*Dothideomyceta*’ [47,48,56]. These intriguing findings increased the interest of researchers concerning the evolution of *Arthoniomycetes*. Indeed, this class was already presented as an intermediate group (“Zwischengruppe”, [57]) due to the ontological development of the ascomata and a multiplicity of morphological traits owned by its representatives. Due to this, several analyses highlighted the importance of including lichen-forming fungi in the *Dothideomycetes* phylogenies [49,58]. In fact, phylogenetic inferences showed that *Arthoniomycetes* was the result of a single, independent lichenization event, and that non-lichenized and lichenicolous species within the class would represent reversions to the non-lichenized state [27,58]. Some years later, Ertz et al. [18] revised the taxonomic relationships between anamorphic and teleomorphic states of lichenicolous species in the genera *Lichenostigma* and *Etayoa*, and recovered these genera next to the *Phaeococcomyces* spp. and *Phaeococcomyces*-like RIF strains/species of Ruibal et al. [54]. The so formed clade was supported as the sister lineage of *Arthoniales* and was formally described as the order *Lichenstigmatales* [18]. Given the position of *Lichenostigmatales* in *Arthoniomycetes*, Ertz et al. [18] stated that the entire order may represent a possible transitional group between *Arthoniomycetes* and *Dothideomycetes*, showing clear affinities with both the lichen-forming fungi in *Arthoniales* and the rock-inhabiting, lichenicolous, and not-lichenized fungi recovered in *Dothideomycetes*. Therefore, the phylogenetic placement of *Antarctolichenia* in *Lichenostigmatales* additionally strengthens the recognition of this order as a key evolutionary connection between the lichen-forming and rock-inhabiting lifestyles in this group of fungi.

In the present study, the connection of *Antarctolichenia* and the entire order *Lichenostigmatales* with the genus *Lichenothelia* is especially crucial to complete the link recurrently hypothesized between rock-inhabiting, meristematic fungi, and lichenized fungi. Indeed, *Lichenothelia* was originally hypothesized to represent “the” link between RIF and lichenized fungi in *Dothideomycetes* [59,60]. However, due to the unclear morphological separation from the strictly lichenicolous genus *Lichenostigma*, some species of either genera were placed together in the family *Lichenotheliaceae* [61]. When Ertz et al. [18] clarified the phylogenetic placement of *Lichenostigma*, including the generic type and other species, they also performed detailed morphological analyses and stated that *Lichenostigma* is distinguishable from the *Lichenothelia* species, by the way the cells divide. The cells in *Lichenostigma* are spherical and multiply by “budding”, instead of by the division through the formation of septa, as in *Lichenothelia*, whose hyphae are clearly filamentous [18,28]. In addition, the phylogenetic inference of Ertz et al. [18] recovered samples of *Lichenothelia* (i.e., corresponding to the formally recognized *Lichenotheliaceae*, *Lichenotheliales* [62]) at the base of *Arthoniomycetes* and as the most basal lineage of the several *Dothideomycetes* orders. The position of the order *Collemopsidiales*, introduced by Pérez-Ortega et al. [32] a few years later, and the description of *Lichenostigmatales* to accommodate the species of the ascomycetous genus *Collemopsidium* (*Xanthopyreniaceae*), also confirmed the evolution of primitive lichen-like associations at the base of *Dothideomycetes*. Simultaneously, this further suggests that the multiple basal lineages in *Dothideomyceta*, which bear the potential to generate borderline forms of lichenization, still lack that particular fungal–algal interdependence characteristic of the lichen symbioses. *Collemopsidiales* was first described as a sister lineage of *Arthoniales* within *Dothideomyceta* [32], while in our analysis it is recovered nested with the *Dothideomycetes* outgroups. Most *Collemopsidium* species, such as *Lichenostigma*, *Lichenothelia*, and *Antartctolichenia*, associate with photosynthetic algae. *Collemopsidium* comprises saxicolous taxa, many of which are rocky seashore dwelling species [63]. Those which associate with cyanobacteria form simple inconspicuous thalli interpreted as borderline lichens [64], one species grows on lichen cephalodia (*C. cephalodiorum*) and another on brown seaweed (*C. pelvetiae*). 

Considering the potential of *Antarctolichenia* to grow and interact with algae (see below), and its phylogenetic position in *Lichenostigmatales*, we may infer two different hypothesis: (i) this lineage may represent a very primitive form of lichenization that may have arisen from an original rock-inhabitant ancestor, as in *Phaeococcomyces*, but has neither evolved further into lichens (as best exemplified by *Arthoniales*) nor into specific parasitic interactions, as in the cases of *Lichenostigma* and *Etayoa*; and (ii) *Antarctolichenia* may represent a half way transition from a lichenized to a free-living lifestyle, still not completely exempt from the relation with algae, since, apparently, they cannot subsist in axenic cultures for a long time. The rise of loose relations with symbiont algae, promoted to adapt and exploit the endolithic niche, may have boosted the transition.

### 5.2. Morphological Traits and the Fungal–Algal Association in Lichenostigmatales

The mycelium structure and lifestyles/ecologies in fungi are strictly correlated, as the mycelium is the feeding and interaction interface of the fungus, modulating the way individuals explore the substrate and the surrounding environment [65]. Only a few fungal species have the capacity to switch between two morphologies, i.e., building either a filamentous or a yeast-like mycelium, and are therefore recognized as dimorphic fungi. Some dimorphic fungi are melanized, microcolonial fungi (so-called “black yeast”), presenting thick cell walls; additionally, they may shift to meristematic growth, traits that confer to them polyextremotolerance [66]. In these fungi, the switch between the two growth forms is triggered by environmental stress factors (i.e., temperature variation, radiation, drought, and anaerobic conditions) or by the capacity to develop virulence and pathogenicity towards their hosts, either plants or animals [67,68]. For species living under permanent stress, meristematic growth may become a stable characteristic. The transition across different growth forms is often a species-specific process in dimorphic fungi, and involves changes in the composition of the cell walls coupled with the thermotolerance of the fungus, sexual reproduction (present in the mycelial phase), and dissemination of conidiospore [67].

The formation of mycelial and yeast-like stages is a phenomenon also documented for representatives of *Arthoniomycetes*. Indeed, species of *Arthoniales* exist in the mycelial stage (i.e., the apical growth of the hyphae and formation of septa between cells) and hyphae are seldom melanized. On the contrary, in *Lichenostigmatales*, in particular in the *Lichenostigma* species, the cells forming the conidiomata and ascomata are melanized, spherical, and multiply by budding, resembling a yeast-like stage, while only asci, ascospores, and conidiogenous cells are not spherical [18]. The ascospores are the only cells possessing real septa but the formation of a mycelial stage was rarely observed; alternatively, conidia were observed to germinate exclusively by budding [18]. Furthermore, the dense and organized agglomeration of yeast cells that form ascomata and conidiomata were reported as a unique characteristic amongst fungi for both the genera *Lichenostigma* [18,69] and *Lichenothelia* [29,70]. Instead, in *Phaeococcomyces*, the sister genus of *Lichenostigma* comprising black yeasts, reproduction is exclusively enacted by budding and no agglomerations are reported [18]. In this study, we observed that also *Antarctolichenia* presents either a filamentous or a yeast-like growth and that this is independent from the culture medium and the isolated strains (Figure 4). Unfortunately, we could not ascertain which growth morphology is developed inside rocks under natural conditions, as the fungus is strictly endolithic and outgrew from rock fragments deposited on an agar medium. While in culture, the yeast-like stage with heavily melanized budding cells is well observable in *Lichenostigmatales* and *Lichenothelia* spp. [29,33,71]; however, ascomata were never observed in neither taxa, so far.

Melanization is known to make fungal cell walls more rigid and less flexible, and coupled with the yeast-like growth, it seems to hinder the formation of haustorium-like hyphae with which the fungus could enwrap or build tight contact with algal cells. However, because melanized fungi, in general, share traits of stress tolerance that allow them to survive in oligotrophic and extremely dry environments, their association with microscopic algae was suggested to potentially improve the meager carbon supplies present in the environment [66]. A borderline lichen symbioses was already observed from some divergent lineages of black fungi in *Dothideomycetes* [29,47,48,49,59,71]; it is also observed in with *Antarctolichenia*, being often isolated together with algal cells. Indeed, two species of melanized *Dothideomycetes* (i.e., *Cystocoleus ebeneus* and *Racodium rupestre*, *Capnodiales*) are the only representatives of well-established lichen symbioses. Their rather primitive thallus, relatively simple in structure, is made by a tight fungal coat of one cell layer around the filamentous photobiont, a thread of *Trentepohlia*, which does not differ much from the free-living forms of the individual symbionts [72]. Other melanized, filamentous, and yeast-like fungi discovered to co-grow in nature in a tight association with algae were used to attempt in vitro co-culture experiments to study their interaction [71,73,74]. The two RIF genera, *Lichenothelia* and *Saxomyces*, are usually found on rocks together with coccoid *Trebouxiophyceae* algae and can be co-grown with them in culture [71]. However, with their phenotypically more plastic mycelia (which develop either filamentous or short yeast-like cells), they are unable to form any clear thallus structures [33,71]. The same behavior was also observed in the halophilic black yeast *Hortaea werneckii*, which was found to co-grow with the algae *Dunaliella atacamensis* on spiderwebs [75]; nevertheless, attempts to co-grow and study this association in vitro culture failed [74]. A certain degree of interaction was also observed by co-culturing some *Coniosporium* or *Knufia* RIF species, where a slight increase in hyphal branching near algal cells was reported [76].

*Antarctolichenia* grows in nature within the rocks together with the algae, as the two bionts were isolated together from the same cell clumps. Though similar to other melanized fungi co-growing with algae, the photosynthetic partners for *Antarctolichenia* seem to be supplementary and not essential for its survival, at least temporarily. Indeed, strains isolated axenically present the same growth development as those in co-culture with the algae, but the axenic culture can be propagated for a limited time only and invariably extinguished if repeatedly subcultured. The perpetuation of pure cultures can be guaranteed by cryopreservation. The type of fungal-algal co-growth observed for *Antarctolichenia*, *Hortaea*, *Lichenothelia*, and *Saxomyces* recalls forms of mycophycobioses, in which fungi are immersed in unchanged colonies of algae without any sign of structural integration. In all these fungi, the development of either the filamentous or the yeast-like mycelium is not concerted with the growth of the algal colony and hyphae or budding cells, respectively, do not form any clear texture in which algal cells are hosted. Nevertheless, these fungal–algal associations represent pivotal springboards for the more specific, stepwise transition that was suggested to have led to the typical lichen symbioses that are now understood as self-sustaining ecosystems [77], in which an ex-habitant fungal partner (the mycobiont) forms a covering structure of hyphal cells embedding the photosynthetic partner (the photobiont) in an extracellular matrix of polysaccharides [78,79].

Sequence analyses and morphological inspections identified the algae associated with *Antarctolichenia* as a *Stichococcus* species, highly similar, genetically, to *S. bacillaris* and closely related to *S. antarcticus*. These results, still preliminary and based on two algal isolates, let us exclude the fact that these algae may introduce contamination during the isolation protocol. *Stichococcus*-like algae are very common and can be found in almost all types of habitats, either aquatic or terrestrial ([36] and references therein). *S. bacillaris* has also been recovered as a secondary photobiont in lichen thalli [80], but, to date, no lichen symbioses are known to share it as a principal photosynthetic partner. This allows us to hypothesize that the species may not be a suitable algal partner for lichenized fungi, and it would be worthwhile to search for other algae as potential photobionts in Antarctic communities. As a detailed analysis of this “potential photobiont” goes beyond the scope of this study, due to the small number of samples available to date, we refrain from further discussing its identity and its capacity to build more or less specific relationships with *Antarctolichenia*. Forthcoming analyses will shed more light on this new fungal–algal partnership.

## 6. Conclusions

Antarctic endolithic communities represent a unique model to address general questions concerning evolutionary ecology given their very simple and stable organization, the strong genetic and geographic isolation, the severe biological constraints imposed by the polar environment that promote adaptive radiation, and the absence of interaction with higher organisms. One of the primary concerns in mycology is to uncover the evolution of lichen symbiotic lifestyles and their role in the diversification of ascomycetes. The new fungal genus and species described in this study further contributes to on the understanding of this evolutionary process. In fact, *Antarctolichenia onofrii*, albeit clearly free-living, still maintains a loose relation to and interdependence with algae, being unable to propagate in axenic culture for a long time; furthermore, it belongs to *Lichenostigmatales*, whose representatives have affinities both with the lichen-forming fungi in *Arthoniales* and the rock-inhabiting, lichenicolous, and not-lichenized fungi in *Dothideomycetes.* Thus, it additionally strengthens its key evolutionary and connective role between the lichen-forming and rock-inhabiting lifestyles.

## Figures and Tables

**Table 1:** Details of the geographic origin and NCBI accession numbers of Antarctolichenia onofrii analyzed in this study. The type strain is highlighted in bold. SVL = southern Victoria Land and NVL = northern Victoria Land. * T = type strain.

Species	MNA-CCFEE	Rock Type	Location	Coordinates		GenBank Accessions	
					nucITS	nucLSU	nucSSU
*Antarctolichenia onofrii*	5284	Sandstone	Battleship Promontory, SVL	−76.90000, 160.90000	MW991415	MW991430	MZ005695
*Antarctolichenia onofrii*	6076	Sandstone	Stewart Heights, NVL	−73.490556, 163.912222	MW991424	MW991432	MZ005699
*Antarctolichenia onofrii*	6093	Granite	Random Hills, NVL	−74.103056, 164.381389	MW991413	MZ005702	MZ005688
*Antarctolichenia onofrii*	6101	Granite	Starr Nunatak, NVL	−75.898889, 162.593889	MW991414	MZ005690	MZ005689
*Antarctolichenia onofrii*	6102 (=MUT 6405)	Granite	Starr Nunatak, NVL	−75.898889, 162.593889	MW991425	MZ005700	MZ005696
*Antarctolichenia onofrii*	6108	Granite	Archambault Ridge, NVL	−73.740556, 162.675556	MW991426	MZ005691	MW989536
*Antarctolichenia onofrii*	6112	Granite	Mt. McGee, NVL	−74.002778, 164.482222	MW991411	MZ005694	MW989537
*Antarctolichenia onofrii*	6161	Sandstone	Mt. Howard, NVL	−75.680556, 161.270833	MW991410	MZ005698	-
*Antarctolichenia onofrii*	6163	Granite	Olson Nunatak, NVL	−75.931944, 162.402222	MW991412	MZ005687	-
*Antarctolichenia onofrii*	6165	Granite	Harrow Peak, NVL	−74.075833, 164.808889	MW991423	MZ005686	MW989728
*Antarctolichenia onofrii*	6294	Granite	Vegetation Island, NVL	−74.784167, 163.659722	-	MZ005701	MW989729
***Antarctolichenia onofrii***	6564 (=MUT 6552) * T	**Sandstone**	**Helliwell Hills, NVL**	**−71.731667, 161.375667**	**MW991427**	**MZ005697**	**MW989731**
*Antarctolichenia onofrii*	6574	Sandstone	Helliwell Hills, NVL	−71.793533, 161.995717	MW991431	MZ005692	MW989732
*Antarctolichenia onofrii*	6583	Sandstone	Helliwell Hills, NVL	−71.793533, 161.995717	MW991428	MZ005693	MW989733

## Data Availability

The datasets supporting the conclusions of this article are available in the NCBI GenBank and the accession numbers are listed in Table 1.

## Supplementary Materials

The following materials are available online at https://www.mdpi.com/article/10.3390/jof7110935/s1. Supplementary Table S1. Fungal taxa selected for the phylogenetic analyses of Figure 2 and Figure S1 are reported with their species name (if present), their ID number, and the corresponding NCBI accessions for the sequences of the ribosomal nuclear large (nucLSU) and small (nucSSU) subunits. Supplementary Table S2. Algal taxa selected for the phylogenetic analyses in Figure S2 are reported with their species name (if present), their ID number, and the corresponding NCBI accessions for the ribosomal nuclear large subunit (nucLSU) and the ribulose 1,5-biphosphate carboxylase large subunit (rbcL) sequences. Supplementary Figure S1. Maximum likelihood phylogenetic analysis based on the nucLSU (A) and plastidial rbcL (B) sequences of the algae *Stichococcus* sp. co-growing with *Antarctolichenia onofrii* isolated strains. (C) Morphology of the algae in light microscopy mounted in water. Supplementary Figure S2. Maximum likelihood phylogenetic analysis based on the nucLSU (A) and plastidial rbcL (B) sequences of the algae Stichococcus sp. co-growing with Antarctolichenia onofrii isolated strains. (C) Morphology of the algae in light microscopy mounted in water.

## Abbreviations

ITS	Internal Transcribed Spacer
MEA	Malt Extract Agar
ML	Maximum Likelihood
MNA-CCFEE	National Antarctic Museum-Culture Collection of Fungi from Extreme Environments
MUT	Mycotheca Universitatis Taurinenesis
NCBI	National Center for Biotechnology Information
nucITS	nuclear Internal Transcribed Spacers
nucLSU	nuclear ribosomal Large SubUnit
nucSSU	nuclear ribosomal Small SubUnit
PCR	Polymerase Chain Reaction
PSFR	Potential Scale Reduction Factor
RIF	Rock Inhabitant Fungi
TM	Trebouxia Medium
