# Supplementary material for: Antarctolichenia onofrii gen. nov. sp. nov. from Antarctic Endolithic Communities Untangles the Evolution of Rock-Inhabiting and Lichenized Fungi in Arthoniomycetes
Source: J Fungi (Basel). 2021 Nov 3;7(11):935. doi: 10.3390/jof7110935 (PMC8621061; doi:10.3390/jof7110935)
Supplement: Supplementary file 1 [file jof-07-00935-s001.zip › Supplementary_Table_S1_rev.pdf]

		NCBI accessions			
**Species name**	**sample ID (LSU/ SSU/ ITS)**	**LSU**	**nuSSU**	**ITS**	**NOTES**
Alyxoria mougeotii	LD:L10058	KJ851078	-	-	type: *Alyxoria diaphora*, no nucLSU and nucSSU sequences available
Alyxoria ochrocheila	Ertz 7500	EU704101	-	-	
Alyxoria subelevata	Ertz 10727	HQ454575	-	-	
Alyxoria varia (1)	Frisch 11-Se1	KJ851027	-	-	
Alyxoria varia (2)	Ertz 7570	EU704103	-	-	
Arthonia caesia	AFTOL 775	FJ469668	-	-	type: *Arthonia radiata*, no nucLSU and nucSSU sequences available
Arthonia cinnabarina	Tehler 9317	HQ454509	-	-	
Arthonia didyma	Ertz 7587	EU704083	-	-	
Arthonia pruinata	Tehler 9139	HQ454510	-	-	
Arthonia punctiformis	Thor 26158	KJ851044	-	-	
Arthothelium galapagoense	Ertz 11790	HQ454516	-	-	type: *Arthothelium scandinavicum*, no nucLSU and nucSSU sequences available
*Botryosphaeria dothidea**	AFTOL 946 / CBS 115.476/ CBS 115.476	DQ678051	DQ677998	KF766151	
Chrysothrix chrysophthalma	Ertz 10927	HQ454519	-	-	type: *Chrysothrix noli-tangere*, no nucLSU and nucSSU sequences available
Collemopsidium angermannicum	s1473 (2Collemops)	KU556871	KU556972		type: *Collemopsidium iocarpum*, no nucLSU and nucSSU sequences available
Collemopsidium pelvetiae	RO25 (1Collemops)	KU556868	KU556965		
Collemopsidium sublitoralis	s464 (8Collemops)	KU556893	-		
Collemopsidium sp.	RO26 (3Collemops)	KU556869	KU556966		
Collemopsidium sp.	s1471 (4Collemops)	KU556975	KU556970		
Collemopsidium sp.	s1472 (5Collemops)	KU556976	KU556971		
Collemopsidium sp.	RO28 (6Collemops)	KU556974	KU556968		
Collemopsidium sp.	RO27 (7Collemops)	-	KU556967		
Collemopsidium sp.	s245 (9Collemops)	KU556875	-		
Collemopsidium sp.	s450 (10Collemops)	KU556882	-		

Collemopsidium sp.	s465 (11Collemop)	KU556889	-		
Collemopsidium sp.	s463 (12Collemop)	KU556887	-		
Collemopsidium sp.	s461 (13Collemop)	KU556886	-		
Coniocarpon cinnabarinum (1)	Frisch13Jp128	MG201844	-	-	type: *Coniocarpon cinnabarinum*
Coniocarpon cinnabarinum (2)	Frisch13Jp127	MG201845	-	-	
Coniocarpon cinnabarinum (3)	Frisch 11Ug196	KP870143	-	-	
Cryptothecia candida	Ertz 9260	HQ454520	-	-	type: *Cryptothecia subnidulans*, no nucLSU and nucSSU sequences available
Cryptothecia sp. (1)	Frisch 11-Ug194	KJ851058	-	-	
Cryptothecia sp. (2)	Ertz 8472	HQ454521	-	-	
Dendrographa alectoroides	-	EF081384	-	AF066941	type: *Dendrographa leucophaea* (not included)
Dendrographa austrosorediata	27936	KY986703	-	KY986702	
Dendrographa decolorans (1)	Frisch11-Se28/ -/ S-F66053	KJ851054	-	KF036005	
Dendrographa decolorans (2)	Ertz 14008	KJ524281	-	-	
Dendrographa decolorans (3)	Ertz 13826	HQ454614	-	-	
Dendrographa leucophaea f. *minor*	missing ID/ missing ID/ Tehler 7680	AF279382	AF043915	AF066957	
*Devriesia strelitziae**	CBS 122.379	-	EU436764	EU43676 3	
Dictyographa arabica	Ertz 11678	HQ454570	-	-	type: *Dictyographa arabica*
Dictyographa varians	Tehler 9346	HQ454576	-	-	
Dimidiographa graphidiza	Tehler 9731	HQ454572	-	-	type: *Dimidiographa loandensis*
Dimidiographa loandensis	Ertz 11686	HQ454573	-	-	
Dimidiographa longissima	Ertz 9155	EU704097	-	-	
Dirina paradoxa (1)	Tehler 10113-03	KC108409	-	KC108020	type: *Dirina ceratoniae* (not included)
Dirina paradoxa (2)	Tehler 10113-04	KC108410	-	KC108021	
Enterographa anguinella	Ertz 10027	EU704086	-	-	
Enterographa cf. *tropica*	Ertz 14196	KJ524292	-	-	
Enterographa crassa	Ertz 5041	EU704088	-	-	type: *Enterographa crassa*
Enterographa hutchinsiae	Ertz 10066	EU704089	-	-	
Enterographa pitardii	Ertz 16854	KJ524290			

Enterographa sorediata	Sanderson 2201/ -/ Sanderson 2328	MF737099	-	MF73709 0	
Etayoa trypethelii (1)	Mukherjee	KF176943	-	-	type: *Etayoa trypethelii*
Etayoa trypethelii (2)	Common 9434K	KF176942	-	-	
Etayoa trypethelii (3)	Common 9215P	KF176941	-	-	
Etayoa trypethelii (4)	Common 9200G	KF176940	-	-	
*Friedmanniomyces endolithicus**	CCFEE 670	GU250366	GU250322	JN885542	
Fulvophyton desertorum	Tehler 9892	HQ454624	-	-	type: *Fulvophyton stalactinum*
Fulvophyton murex	Tehler 9191	HQ454627	-	-	
Fulvophyton rostratum	Tehler 9184_77	HQ454630	-	-	
Fulvophyton stalactinum	Tehler 9902	HQ454518	-	-	
Fulvophyton subseriale	Tehler 9314_19	HQ454530	-	-	
Ingaderia pulcherrima (1)	Tehler 9886	HQ454539	AF043919	-	type: *Ingaderia pulcherrima*
Ingaderia pulcherrima (2)	Tehler 9896	HQ454540	-	-	
Lecanactis borbonica	Ertz 17876	KJ524302	-	-	type: *Lecanactis abietina*
Lecanactis epileuca	Ertz 15561/ -/ missing ID	KJ524303	-	AF138836	
Lecanactis submollis	Ertz 7995	EU704091	-	-	
Lecanographa amylacea	CCFEE 5922	KR781040	-	-	type: *Lecanographa lyncea*
Lecanographa atropunctata	Ertz 9869	HQ454548	-	-	
Lecanographa brattiae	Ertz 12437	HQ454571	-	-	
Lecanographa farinosa cf.	Ertz 14042	HQ454546	-	-	
Lecanographa dialeuca	Ertz 10569	HQ454550	-	-	
Lecanographa dimelaenoides	Tehler 9109	HQ454551	-	-	
Lecanographa hypothallina	Tehler 9092	HQ454557	-	-	
Lecanographa lyncea	Tehler 9123	HQ454560	-	-	
Lecanographa uniseptata	Ertz 9859	HQ454561	-	-	
Lichenostigma alpinum (1)	Ertz 17519	KF176946	-	-	type: *Lichenostigma maureri*
Lichenostigma alpinum (2)	Ertz 17522	KF176945	-	-	
Lichenostigma chlaroterae (1)	Neuberg/Diederich	KF176948	-	-	
Lichenostigma chlaroterae (2)	Goward 12-42	KF176949	-	-	

Lichenostigma maureri (1)	Diederich 17326	KF176953	-	-	
Lichenostigma maureri (2)	Diederich 17337	KF176954	-	-	
Lichenostigma maureri (3)	Diederich 17306	*KF176951*	-	-	
Lichenostigma sp. (1)	Diederich 17240	KF176955	-	-	
Lichenostigma sp. (2)	Ertz 17591	KF176961	-	-	
*Mycosphaerella graminicola**	AFTOL 1615	DQ678084	DQ678033	-	
*Mytilinidion resinicola**	CBS 304.34	NG_057807	FJ161145	MH85553<br>5	
Paraingaderia placodioidea (1)	Tehler 9348	HQ454634	-	-	type: *Paraingaderia placodioidea*
Paraingaderia placodioidea (2)	Tehler 9315	HQ454632	-	-	
Paralecanographa grumulosa (1)	Ertz 13568	HQ454556	-	-	type: *Paralecanographa grumulosa*
Paralecanographa grumulosa (2)	Tehler 9809	HQ454542	-	-	
Paralecanographa grumulosa (3)	Tehler 9031	HQ454553	-	-	
Paraschismatomma ochroleucum (1)	Tehler 9089	HQ454619	-	-	type: *Paraschismatomma ochroleucum*
Paraschismatomma ochroleucum (2)	Ertz 12421	HQ454615	-	-	
Paraschismatomma ochroleucum (3)	Tehler 9118	HQ454616	-	-	
Pentagenella fragillima	-	EF081395	GU323991	-	type: *Pentagenella fragillima*
Pentagenella gracillima	Tehler 9897	HQ454536	-	-	
Pentagenella sp.	Tehler 8916	HQ454577	-	-	
*Petrophila incerta**	TRN 77	GU323963	GU323993	-	
Phaeococcomyces eucalypti	CPC:17606	KC005791	-	-	type: *Phaeococcomyces nigricans*
Phaeococcomyces nigricans (1)***T**	CBS 652.76	AF361048	-	-	
Phaeococcomyces cf. *nigricans* (2)	MZ107	AJ276065	-	-	
Phaeococcomyces sp. TRN452	TRN452 - *P. eucalypti* ?????	GU323985	GU324014	AY843150	
Phaeococcomyces sp. TRN456	TRN456 - *P. nigricans* ???	GU323986	GU324015	AY843154	

Phaeococcomycetaceae sp. TRN529	TRN529	GU323987	GU324016	AY843200	
Phaeococcomycetaceae sp. TRN213	TRN213	-	GU324008	AY843079	
*Preussia terricola**	AFTOL 282/ AFTOL 282 / CBS 317.65	AY544686	AY544726	MH858589	
Reichlingia leopoldii (1)	Ertz 13294	HQ454582	-	-	type: *Reichlingia leopoldii*
Reichlingia leopoldii (2)	Ertz 13293	HQ454581	-	-	
Roccella hypomecha	missing ID/ missing ID/ S-L2985	EF081438	AF138842	KF036017	
Roccella lirellina (1)	Tehler 8880	FJ639015	-	EU449898	
Roccella lirellina (2)	Tehler 8904	FJ639016	-	EU449903	
Roccella lirellina (3)	Tehler 8919	FJ639017	-	EU449904	
Roccella phycopsis (1)	S-F205996/ - /8148(S)	KF036036	-	AJ634027	
Roccella phycopsis (2)	missing ID /- / 8150b(S)	EF081423	-	AJ634029	
Roccella sinensis (1)	Tehler 8571	GU138023	-	GU137905	
Roccella sinensis (2)	Tehler 8564	GU138022	-	GU137904	
Roccellina cerebriformis (1)	Tehler 8386/ - / missing ID	HQ454585	-	EF081377	type: *Roccella fuciformis*
Roccellina cerebriformis (2)	Tehler 8387/ - / S-L28663	HQ454586	-	KF036013	
Roccellina mahuiana	Tehler 8459	HQ454596	-	KF036020	
Roccellina terrestris	Tehler 9917/ - / S-F206063	HQ454598	-	KF036023	
Roccellographa circumscripta (1)	Ertz 14055	HQ454580	-	-	type: *Rocellographa cretacea*
Roccellographa circumscripta (2)	Ertz 13872	Ertz 14055	-	-	
Roccellographa cretacea (1)	AFTOL 93	DQ883696	DQ883705	AF138825	
Roccellographa cretacea (2)	Tehler 9313	HQ454599	-	-	
Roccellographa cretacea (3)	Tehler 9324	HQ454600	-	-	

*Saxomyces alpinus**	CCFEE 5491	KC315874	KC315863	-	
Schismatomma dirinellum (1)	S-F206034	KF036039	-	*KF036025*	type: *Schismatomma pericleum*
Schismatomma dirinellum (2)	Diederich 16390	HQ454613	-	-	
Schizopelte californica	Tehler 9082/ Tehler 9082/ Tehler 7526	HQ454620	AF138847	AF138828	type: *Schizopelte californica*
Schizopelte crustosa (1)	Tehler 9155	HQ454564	-	-	
Schizopelte crustosa (2)	Ertz 12416	HQ454562	-	-	
Schizopelte parishii	Tehler 9107	HQ454533	-	-	
Sigridea californica (1)	Ertz 12456/ - / Tehler 7510	HQ454635	-	AF138830	type: *Sigridea californica*
Sigridea californica (2)	Ertz 12491/ - /Tehler 7407	HQ454636	-	AF138829	
Sparria cerebriformis (1)	Tehler 9156	HQ454568	-	-	type: *Sparria cerebriformis*
Sparria cerebriformis (2)	Tehler 9097	HQ454565	-	-	
Sparria endlicheri	Ertz 14068	HQ454513	-	-	
Syncesia intercedens	Ertz 11059	HQ454644	-	MF73709 1	type: *Syncesia albida*, no nucLSU and nucSSU sequences available
Syncesia mascarena	Ertz 17987/ - / Ertz 18052	KJ524334	-	MF73709 2	
Syncesia myrticola	Ertz 16273	KJ524336	-	MF73709 4	
Syncesia socotrana	Tehler 9347	HQ454531	-	-	
Zwackhia sorediifera	Thor 26210	KJ851055	-	-	type: *Zwackhia involuta*, no nucLSU and nucSSU sequences available
Zwackhia viridis	Ertz 7619	EU704106	-	-	
