# Supplementary material for: Antarctolichenia onofrii gen. nov. sp. nov. from Antarctic Endolithic Communities Untangles the Evolution of Rock-Inhabiting and Lichenized Fungi in Arthoniomycetes
Source: J Fungi (Basel). 2021 Nov 3;7(11):935. doi: 10.3390/jof7110935 (PMC8621061; doi:10.3390/jof7110935)
Supplement: Supplementary file 1 [file jof-07-00935-s001.zip › Supplementary_Table_S2.pdf]

NCBI accession

Species Name	Strain ID	LSU	rbcL
Chaetophora elegans	ACOI 457	HF920663	-
Chaetophora pisiformis	XR201704	MH002628	-
Chaetophora sp.	BEA 0173B	LN870282	-
Characiochloris acuminata	UTEX 2095	AF395493	-
Chlamydomonas frankii	SAG 18.72	AF395499	-
Chlamydomonas pulsatilla	UTEX 410	DQ015732	-
Chlorella ellipsoidea	-	D17810	-
Chlorella pyrenoidosa	strain 820	-	EU038284
Chlorella sorokiniana	CS-11_35-2	KX639541	-
Chlorella variabilis	KGE28	HE974916	-
Chlorella vulgaris	YSR014	FR751204	-
Chlorella vulgaris	YSR023	FR751202	-
Chlorella vulgaris	KNUA007	KJ148625	-
Chlorella sp. ('Chlorella-like')	-		M74441
Chlorosarcina stigmatica	UTEX 962	DQ015738	-
Coccomyxa sp.	sp. Obi	MK694759	-
Coccomyxa sp.	sp. KJ	MK694758	-
Coccomyxa sp.	OTU 15	KX383957	-
Coccomyxa sp.	L599	-	HQ287488
Coccomyxa sp.	MW8233n clone L629	-	HQ287504
Coccomyxa sp.	MW8233p clone L630	-	HQ287505
Coccomyxa sp.	PL3-1-3b clone L570	-	HQ287482
Coccomyxa sp.	PL3-2-3a clone L571	-	HQ287483
Coccomyxa simplex	SAG 216-10	-	JF502545
Coccomyxa simplex	SAG 216-11a	-	JF502546
Coccomyxa subellipsoidea		KX383958	-
Diplosphaera sp.	CG831	-	JN573835
Diplosphaera sp.	CG601	-	JN573825
Diplosphaera sp.	L515	-	JN573848
Diplosphaera sp.	SAG 49.86	-	AM260445
Diplosphaera chodatii	SAG 2.82	-	LR777825
Drapalnardia glomerata	CCAP 418/2	HF920670	-
Eremosphaera viridis	CCAC 0071	HE610127	-
Ettlia carotinosa	SAG 213-4	KR181935	-
Gongrosira papuasica	UTEX 1916	DQ015756	-
Heterochlamydomonas inaequalis	UTEX 1705	AY206708	-
Heterochlamydomonas rugosa	SAG 45.86	AY206709	-
Koliella longiseta	SAG 470-1	HE610126	-
Lobocharacium coloradoense	strain Kugrens	AF395509	-
Micractinium reisseri	-	AB506071	-
Micractinium reisseri	-	AB437244	-
Mychonastes zofingiensis	UTEX 56	KC145443	-
Nannochloris normandinae	SAG 9.82	-	JF502540
Oogamochlamys zimbabwiensis	UTEX 2213	DQ015729	-
Oogamochlamys zimbabwiensis	UTEX 2214	DQ015758	-
Prasiola crispa	SAG 43.96	AM260453	-
Prasiola furfuracea	-	-	AF189064
Prasiola linearis	-	-	AF189065
Prasiola meridionalis	-	-	AF189066

Pseudochlorella pringsheimii	RSN07	MH716039	-
Pseudochlorella pringsheimii	strain VIT_SDSS	MG889861	-
Pseudochlorella pringsheimii	-	KY364701	-
Pseudochlorella pringsheimii	-	MK433636	-
Pseudostichococcus monallantoides	UTEX 2249	-	LR777828
Schizomeris leibleinii	UTEX LB 1228	AF183483	-
Sphaeropleales sp. KF-2013a	BCP-CC1VF5A	KC145441	-
Stichococcus allas	ASIB37	-	MH672681
Stichococcus antarcticus	Beck VALK15/01cVI (M) M-0019660	-	MH672667
Stichococcus antarcticus	Beck FiSo15/03cVI (M) M-0019691	-	MH672679
Stichococcus antarcticus	Beck FiSo15/01dIV (M) M-0019690	-	MH672678
Stichococcus antarcticus	Beck VALK15/02dII (M) M-0019684	-	MH672673
Stichococcus antarcticus	Beck FiSo15/03fVI (M) M-0019682	-	MH672671
Stichococcus antarcticus	Beck GreP15/01dI (M) M-0019667	-	MH672655
Stichococcus antarcticus	Beck Dec15/07cII (M) M-0019664	-	MH672653
Stichococcus bacillaris	SAG 379-2	HE610125	-
Stichococcus bacillaris	strain ST-4	-	LR777817
Stichococcus bacillaris	strain ST-3	-	LR777816
Stichococcus bacillaris	strain ST-10	-	LR777815
Stichococcus bacillaris	SAG 2406	-	LR777818
Stichococcus deasonii	SAG 2139	-	LR777811
Stichococcus jenerensis	CAUP J 1302	-	KM438447
Stichococcus sp.	cort02	-	HG793061
Stichococcus sp.	cort05	-	HG793062
Stichococcus sp.	BCP-ZNP2-VF4	-	KC207724
Stichococcus sp.	cort07	-	HG793064
Stichococcus sp. L3841	L3841	xxxxxxxx	xxxxxxxx
Stichococcus sp. L3843	L3843	xxxxxxxx	xxxxxxxx
Stigeoclonium elongatum	CCAC 3494 B	HF920678	-
Stigeoclonium helveticum	UTEX B441	KC817002	-
Stigeoclonium tenue	CCAP 477/11A	HF920680	-
Tetraselmis striata	strain a	KY549667	-
Tetraselmis suecica	MK1	KC415759	-
Trebouxia arboricola	SAG 219-Ia	-	AM158960
Trabouxioa asymmetrica	SAG 48.88	-	AB194860
Trebouxia corticola	Ohmura 5415	-	AB194862
Trebouxia decolorans	P-120a-IIIb	-	AM158966
Trebouxia showmanii	UTEX 2234	-	AB194857
Trebouxia gelatinosa	-	Z95382	-
Trebouxia sp. TR9	-	KU716051	-
Uronema belkae	UTEX 1179	AF183489	-
Uronema trentonense	CCAP 386/5	HF920659	-
