# Supplementary figures and images for: Antarctolichenia onofrii gen. nov. sp. nov. from Antarctic Endolithic Communities Untangles the Evolution of Rock-Inhabiting and Lichenized Fungi in Arthoniomycetes
Source: J Fungi (Basel). 2021 Nov 3;7(11):935. doi: 10.3390/jof7110935 (PMC8621061; doi:10.3390/jof7110935)

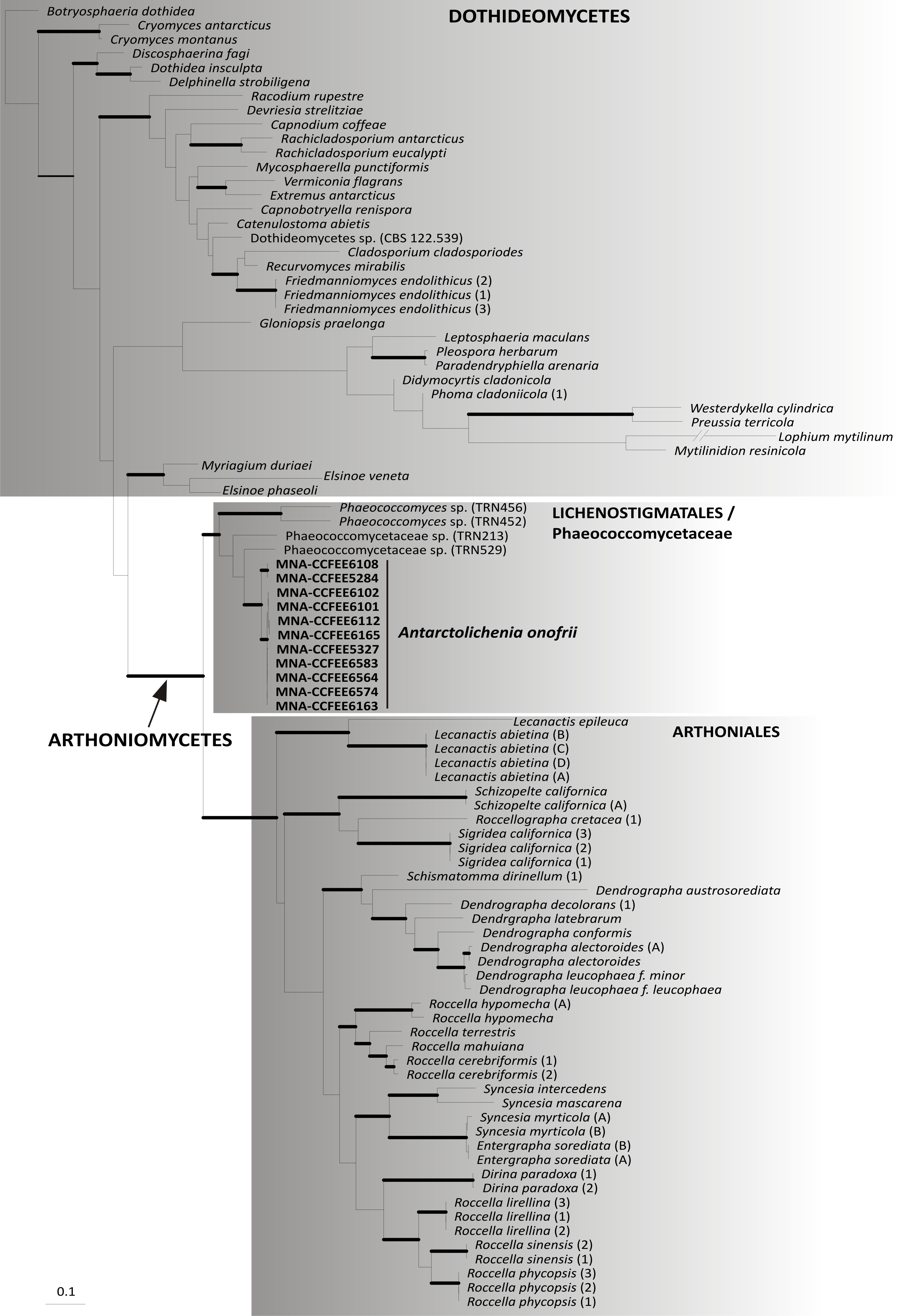

Supplement: Supplementary file 1 [file jof-07-00935-s001.zip › Supplementary_Figure_S1_REV.jpg]

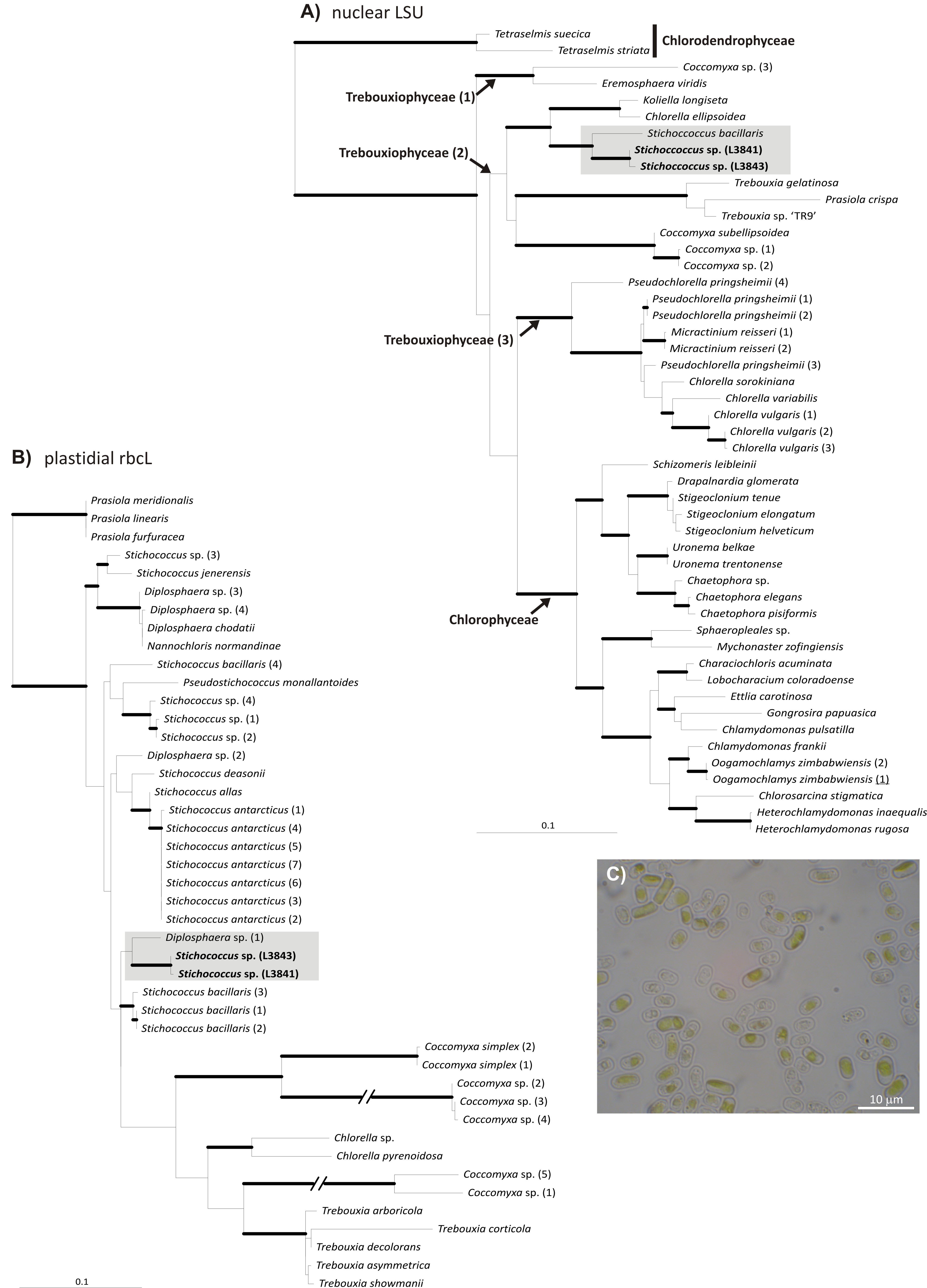

Supplement: Supplementary file 1 [file jof-07-00935-s001.zip › Supplementary_Figure_S2.jpg]
